# Anesthetic Challenges Associated With Posterior Reversible Encephalopathy Syndrome in a Pregnant Woman Scheduled for a Caesarean Section

**DOI:** 10.7759/cureus.53079

**Published:** 2024-01-27

**Authors:** Vivek Chakole, Jui A Jadhav, Shrilekh Mankhair, Sambit Dash

**Affiliations:** 1 Department of Anaesthesiology, Jawaharlal Nehru Medical College, Datta Meghe Institute of Higher Education & Research (Deemed to Be University), Wardha, IND

**Keywords:** posterior reversible encephalopathy syndrome, lscs, sub arachnoid blockage, spinal anesthesia, eclampsia

## Abstract

Recently, there has been a rise in reports of posterior reversible encephalopathy syndrome (PRES), which is an uncommon neurologic illness. The precise cause of PRES syndrome is yet unknown, but there are certain illnesses that have been associated with it. Furthermore, because of advances in imaging methods and growing awareness, the connection between PRES and pre-eclampsia/eclampsia is becoming increasingly recognised. Pre-eclampsia/eclampsia by itself poses distinct perioperative difficulties; in addition, PRES makes anesthesia administration more difficult. Regretfully, there is a lack of knowledge regarding the anesthetic treatment provided to the extremely sick and medically complex patients, and it is uncertain whether the chosen anesthetic might exacerbate neurologic problems. Here, we discuss the implications for the anesthetic management of PRES presentations.

## Introduction

With new developments in technology and imaging modalities, the general population's understanding of posterior reversible encephalopathy syndrome (PRES) is increasing, and mortalities associated with it, mostly due to negligence, are diminishing. Since PRES doesn't have a precise diagnostic criterion, it is regarded as a diagnosis of exclusion [1]. The total incidence is difficult to calculate and probably underestimated; it is less than 1% of at-risk population segments (solid organ transplantation, end-stage renal illness, and systemic lupus erythematosus) [2,3]. Hypertension, sepsis, renal insufficiency, cytotoxic drugs, immunosuppressive treatment, and autoimmune illnesses are among the conditions linked to PRES. Moreover, when neurologic symptoms are present, there is mounting evidence that pre-eclampsia and eclampsia are linked to the diagnosis of PRES [4]. Patients with pre-eclampsia or eclampsia require more complicated anesthetic care because of the many physiological abnormalities related to the gravid state.

## Case presentation

**Figure 1:**
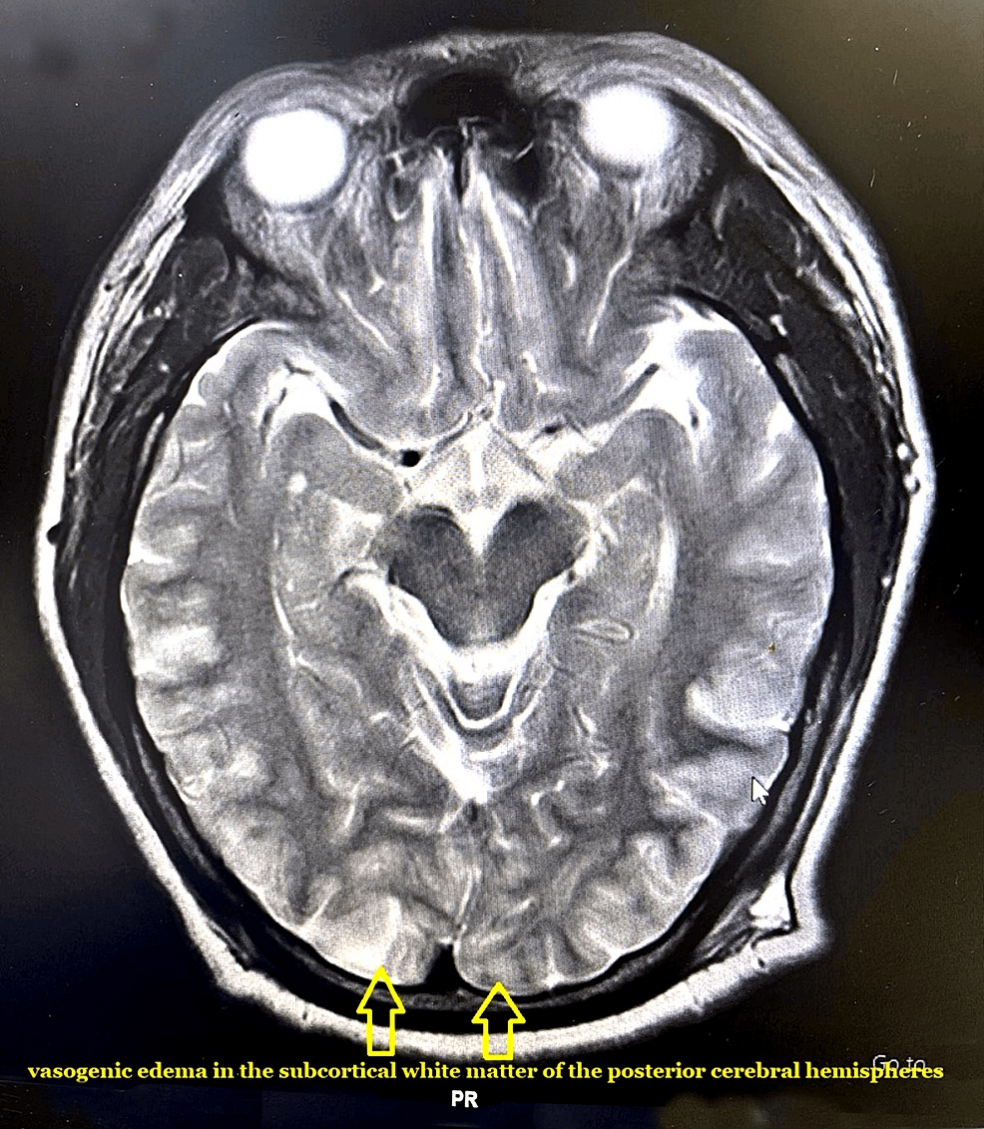
CT image suggestive of posterior reversible encephalopathy syndrome

A 27-year-old primigravida with 28.3 weeks of gestational age presented with a complaint of loss of consciousness and seizure. It was associated with tongue bite and a history of a fall the same morning. The patient was undergoing a routine checkup for pregnancy at a private hospital and at 20 weeks of gestational age, her blood pressure (BP) was found to be elevated at 150/90 mmHg; the patient was put on tablet labetalol 100 mg once daily and tablet aspirin 150 mg once daily. During history taking, it was found that the patient was not adherent to the drug regimen. On examination in the emergency room (ER), patient’s pulse rate (PR) was 86 bpm and BP 200/110 mmHg. On auscultation, chest was found to be clear with no added sounds. Normal heart sounds were heard with no signs of congestive heart failure. The patient was admitted to the critical care unit for the management of gestational hypertension and all baseline laboratory investigations were done. A computed tomography (CT) scan followed by magnetic resonance imaging (MRI) was advised with magnetic resonance angiography (MRA) and magnetic resonance venography (MRV), which was suggestive of posterior reversible encephalopathy syndrome with thin pericallosal lipoma (Figure 1). MRI was advised as it is a more sensitive and definitive diagnostic modality for PRES after initially suspecting changes in the CT scan that pointed towards the probability of PRES. The recommended bedside ultrasound showed one live fetus with a body size commensurate with 28 weeks and a normal amount of amniotic fluid. The patient was diagnosed with eclampsia and had 3(+) proteinuria.

The patient was scheduled for an emergency caesarean section. Her pre-anesthetic checkup (PAC) was done, and her vitals were PR 98 bpm and BP 180/110 mmHg; mouth opening (MO) and Mallampati classification (MPC) score could not be assessed due to patient's altered mental status and noncompliance. The patient was classified under American Society of Anesthesiologists (ASA) classification grade IV indicating severe systemic disorder, which is a constant threat to life. The patient being non-coherent, written and verbal consent was obtained from patient's relative. The patient was sent immediately to the operation theatre (OT) as she was disoriented with regard to time, location, and person. Soon after shifting, inside the OT, multiparameter monitors as per Indian Society of Anesthesiology (ISA) standards were attached, and two large-diameter 16G cannulas for intravenous access were placed. Induction of anesthesia was started with injections of 5 mg/kg of thiopental sodium and 0.5 mg/kg of atracurium given intravenously. Next, sevoflurane at 0.75 minimum alveolar concentration (MAC) and 50% O_2_ + 50% N_2_O were given. A 7.5-mm endotracheal tube was used to intubate the patient. The surgeon was informed to make the incision coinciding with our induction of anesthesia. Through caesarean delivery, one viable female baby was born.

The birth weight of the baby was documented to be 1100 gm and height was measured to be 36 cm with a one-minute APGAR (Appearance, Pulse, Grimace, Activity, and Respiration) score of 7 and a five-minute APGAR score of 9. Postoperatively, the patient was extubated under deep anesthesia in view of cerebral edema and preoperatively raised BP, after the initiation of spontaneous respiration. Postextubation, the patient was monitored in the operating room for 10 minutes and the respiratory pattern was assessed to be normal. After continuous monitoring and observation and maintenance of peripheral oxygen saturation (SpO_2_) on room air, the patient was shifted to the intensive care unit (ICU). In the ICU, her BP increased to 180/110 mmHg. Hence, nitroglycerine (10 mcg/kg/hr) and magnesium sulphate (2 g/hr) infusion was continued. The patient was administered dexamethasone injections (24 mg/day) and was given tablet amlodipine (a calcium channel blocker). Regular follow-ups were done till the patient was haemodynamically stable, and she was shifted out of the ICU on postoperative day 6. A follow-up CT scan done on postoperative day 5 showed improvement in edema. The patient was discharged from the hospital on postoperative day 12.

## Discussion

Headache, altered mental state, convulsion, and visual loss with reversible subcortical vasogenic edema in the posterior cerebral white matter are among the clinical and radiological symptoms that define PRES [5]. PRES has been associated with the following conditions: cytotoxic drugs, autoimmune illnesses, high blood pressure, eclampsia, pre-eclampsia, and kidney disorders. Though much remains to be discovered about the pathophysiology, many mechanism have been proposed. According to the most widely accepted explanation, sudden spikes in the mean arterial pressure interfere with autoregulation, damaging the blood-brain barrier and creating openings in the arteries that allow plasma and macromolecules to enter the cerebral white matter [6].

After 20 weeks of gestation, pregnancy-related hypertension disorders known as pre-eclampsia and eclampsia manifest. Hypertension (systolic blood pressure >140 mmHg and/or diastolic blood pressure >90 mmHg) without exacerbating factors that is associated with one or more of the following is called pre-eclampsia: proteinuria, uteroplacental dysfunction, acute kidney damage, liver involvement, neurologic difficulties, or hematologic issues that are some of the manifestations of maternal organ failure [7].

It is unclear what the ideal anesthetic approach is for PRES given the number of case reports that explicitly connect anesthesia to the illness. Anesthetics and PRES have frequently been associated since there is a temporal correlation and no established starting variable [8]. Dural puncture, as documented in many studies, may be the reason for the development of PRES, but there is not enough evidence to contradict the use of neuroaxial blockade in suspected cases of PRES.

## Conclusions

The pathology and disease development of PRES, which include brain injury, vasogenic edema, and the potential danger of high intracranial pressure, complicate anesthesia planning. The case reported here demonstrates how little is known about treating this illness during pregnancy, while also contributing to the body of knowledge. With recent developments, better affordability of MRI scans and an increase in the number of MRI centres, it is plausible that in the future, healthcare providers may be able to identify and diagnose a higher number of cases with PRES. Further research with a larger population is needed to ascertain the safety and efficacy of the anesthesia technique that can be implemented in patients with suspected PRES.
